# The influence of cardiovascular fitness and ventilatory efficiency on fMRI assessed cerebrovascular reactivity in older adults

**DOI:** 10.3389/fphys.2025.1581187

**Published:** 2025-05-08

**Authors:** Tom S. Novak, Kevin Mammino, Medina Bello, Isabella Paredes Spir, Mark Vernon, Venkatagiri Krishnamurthy, Joe R. Nocera

**Affiliations:** ^1^ Center for Visual and Neurocognitive Rehabilitation, Atlanta VAMC, Decatur, GA, United States; ^2^ Division of Physical Therapy, School of Medicine, Emory University, Atlanta, GA, United States; ^3^ Department of Neurology, School of Medicine, Emory University, Atlanta, GA, United States; ^4^ Atlanta VAMC Division of Rehabilitation Research, Decatur, GA, United States

**Keywords:** cerebrovascular reactivity (CVR), VO₂ max, ventilatory efficiency, VE/VCO2 slope, functional magnetic resonance imaging (fMRI), aging

## Abstract

**Purpose:**

Biological aging has a profound impact on cerebral health. A prevalent factor thought to underpin overall brain health is healthy cerebrovascular function. Recent research suggests a beneficial relationship between cerebrovascular health and physical activity. Specifically, transcranial doppler (TDS) studies have shown that higher hypercapnic cerebrovascular reactivity (CVR) at the major cerebral arteries is associated with better cardiovascular fitness in older adults. Building on this previous literature, we tested the hypothesis that fMRI-based capillary/venous CVR dynamics would also demonstrate a positive relationship with cardiovascular fitness. We also explored whether the magnitude and direction of CVR-fitness relations are consistent across the whole brain or demonstrate regional heterogeneity.

**Methods:**

Twenty-one cognitively intact, older adults aged 65–80 years completed an fMRI-BOLD CVR scan in which individuals alternated between breathing normal room air and a hypercapnic gas mixture (5% CO_2_, 21% O_2_, balanced N_2_). Cortical atlas segmentation and voxel-wise CVR analyses were performed to explore regional specificity of CVR-fitness relations. To quantify cardiovascular fitness, participants were assessed with graded exercise testing where estimated VO_2_ max, VE/VO_2_ slope, and VE/VCO_2_ slope (ventilatory efficiency) outcomes were collected.

**Results:**

In contrast to the TDS literature, our multiple regression analysis found that higher estimated VO_2_ max, and greater ventilatory efficiency (lower VE/VCO_2_ slope) were associated with lower hypercapnic CVR (all *p < 0.03)*. This inverse relation was consistent across all cortical ROI’s, however, estimated VO_2_ max outcomes accounted for considerably more variance in CVR at the frontal, temporal, and parietal ROI’s, while greater ventilatory efficiency (VE/VCO_2_) exhibited a strong relation with lower CVR across the cortex.

**Conclusion:**

This work suggests that cardiovascular fitness is associated with cortical CVR, however, the strength and direction of this relationship may depend largely on the vascular beds being measured. Considering the complex array of physiological mechanisms contributing to BOLD-CVR (I.e., endothelial, glial, mitochondrial function) future studies using multi-modal CVR assessment can further inform the specificity of neural and vascular-based CVR adaptations in the aging brain.

## 1 Introduction

Within the next decade adults over the age of 65 are projected to account for over one-fifth of the global population (Vespa, 2018). This presents a variety of social, economic, and healthcare challenges as physiological declines due to aging increase the incidence of neurological insult that adversely affect cognitive faculties, functional independence, and overall quality of life (Aguilar-Hernandez et al., 2023; Kowalska et al., 2017; Zuo and Wu, 2022). While aging itself is a major risk factor for neurological disease, sedentary behavior further increases this risk due to cardiovascular co-morbidities such as hypertension, atherosclerosis, and stroke (Bai et al., 2024; Ballin et al., 2021; Hooker et al., 2022). Nevertheless, the trajectory towards progressive senescence varies significantly between individuals. To date, increased physical activity and cardiovascular fitness are effective modalities for attenuating neurological disease and associated pathologies. As such, there is a pressing need to better understand the physiological underpinnings that protect brain health and function with exercise so that targeted intervention can be optimized to improve overall health and quality of life in aging (Aguilar-Hernandez et al., 2023; Fragala, 2015; Jackson et al., 2016).

Research has indicated that, intertwined with aging-related declines in brain health and function, are physiological changes in cerebrovascular health. For example, epidemiological and clinicopathological data indicate considerable overlap between cerebrovascular dysfunction and Alzheimer’s disease (Dickstein et al., 2010; Klohs, 2019). In healthy cortices, regulation of blood flow via vasodilation and vasoconstriction are necessary to maintain perfusion according to dynamic metabolic demands and limit hypoxic insult. Established approaches to examining the functional status of cerebrovascular health is measuring cerebrovascular reactivity (CVR), which we define as the capacity for change in cerebral blood flow in response to a vasodilatory/vasoconstrictive stimulus (Fierstra et al., 2013a). One established approaching to examining CVR is through carbon dioxide (CO_2_) inhalation utilizing transcranial doppler ultrasound (TDS) or functional magnetic resonance imaging (fMRI) (Chiarelli et al., 2007; Yezhuvath et al., 2009).

Evidence from the TDS literature suggests that CVR response to a hypercapnic challenge progressively declines across the lifespan (Kastrup et al., 1998; Keage et al., 2012). More specifically, compared to healthy younger adults, older individuals tend to exhibit smaller increases in blood flow velocity at the major cerebral arteries, which is thought to reflect altered function of numerous processes including production of vasodilatory/vasoconstrictive endothelial factors (vasodilatory: nitric oxide (NO), prostacyclin (PGI_2_), endothelium derived hyperpolarizing factor (EDHF); vasoconstrictive: thromboxane (TXA_2_), endothelin-1 (ET-1)) (Toda, 2012), along with structural changes within the major arteries (i.e., loss of smooth muscle elasticity, endothelial fibrosis, increased collagen density/arterial stiffness). Consistent with this assumption, cardiorespiratory fitness has been shown to account for differences in TDS-CVR in older adults, with higher cardiovascular fitness corresponding with greater increases in blood flow velocity at the middle cerebral artery (Barnes et al., 2013). While compelling, this work is limited by the fact that TDS only interrogates large basal arteries and can only provide an index of global rather than local cerebral blood flow velocity.

Blood oxygen level dependent (BOLD) functional magnetic resonance imaging (fMRI) affords whole-brain assessment of aging-related changes in cortical vascular dynamics. CVR is known to vary across the cortex, which may reflect tissue- and region-specific contributions of cerebrovascular control according to distinct functional and structural characteristics. Cortical grey matter exhibits a threefold increase in CVR compared to white matter tissues (Rostrup et al., 2000; van der Zande et al., 2005), and correspondingly, CVR is higher in cortical grey-matter relative to deeper grey matter structures (Novak, 2012), and grey matter CVR is regionally heterogenous in both the young healthy and aging cortices (Catchlove et al., 2018; Kastrup et al., 1999). This work suggests that inter-individual variations in regional CVR may exhibit a direct relationship to functional demand. For example, it is documented that greater temporal and hippocampal CVR predicts better memory performance in otherwise healthy older adults (Catchlove et al., 2018; Kastrup et al., 1999).

Collectively, these findings indicate the significance of interrogating inter- and intra-individual variations in cerebrovascular function, particularly as they relate to physiological and behavioral outcomes that help index the progression of aging-related senescence. There is ample evidence that cardiorespiratory fitness levels exert significant positive effects on brain health and function (Barnes et al., 2003; McGregor et al., 2018; Nocera et al., 2020), grey matter thickness (Olivo et al., 2021a), resting-state cortical blood flow (Olivo et al., 2021b), and task-dependent BOLD activation patterns (McGregor et al., 2018; Nocera et al., 2020; Krishnamurthy et al., 2022). However, to date, the effects of aging and cardiovascular fitness on the regional specificity of CVR remain inconclusive. As such, this study sought to investigate the relation between individual differences in cardiovascular fitness and CVR as assessed by BOLD fMRI. We examined the relationship of whole-brain and regional BOLD-CVR to explore whether the magnitude and direction of CVR-fitness relations are consistent across the whole brain or demonstrate regional heterogeneity. In addition, because CO_2_ is the stimulus for the measured change in CVR, we examined CO_2_ elimination during graded exercise (VE/VCO_2_ slope). We hypothesized a positive relationship between fitness and whole-brain CVR, such that greater reactivity would be positively correlated with higher estimated VO_2_ max and inversely correlated with a lower VE/VCO_2_ slope (greater ventilatory efficiency). We also hypothesize regional consistency of this relationship.

## 2 Methods

### 2.1 Subjects

We report on data collected from 23 participants (age: 70.98 ± 3.9 years, 16 Female) who volunteered for this study. All participants underwent a medical exam from their primary care physician and attained physician signature deeming them safe to undergo exercise testing. All participants were sedentary as defined by not meeting the current ACSM/AHA weekly activity guidelines. All participants reported having no history of neurological disease, stroke, myocardial infarcts, pulmonary disease, or lower/upper-extremity impairments that would impact their ability to complete graded exercise testing. Eight reported using ACE inhibitors for hypertension. Participants were excluded if they were on Beta-blockers and were also screened for any conditions that may be contraindicated for MRI assessment. All participants were provided with informed consent and provided their written consent before any testing commenced. All study procedures complied with the Declaration of Helsinki and were approved by the Emory University Internal Review Board and Atlanta VA Research and Development Office (Grant: I01RX002825-01A2).

Participants were assessed in two separate sessions for this study, both of which occurred within 2 weeks of each other. The first session involved a graded exercise test while the second session involved both anatomical and function MRI.

### 2.2 MRI acquisition

MRI data were acquired via a 3T Siemens Prisma MRI scanner (Erlangen, Germany) using a 32-channel phased-array head coil. The anatomical acquisition was acquired utilizing T1w-MPRAGE: TR = 2,530 m, TE = 3.08 m, FA = 7°, resolution = 0.8 mm isotropic, acquisition bandwidth = 130 Hz)

Our cerebrovascular reactivity (BOLD-CVR) scan utilized a blocked-design fixed CO_2_ inhalation paradigm. Our participants were first familiarized with the scanning environment and positioned comfortably on the scanner bed. Participants were then fitted with a mouthpiece attached to a two-way non-rebreathing valve that was affixed to the scanner bed using a custom 3D printed gooseneck. Connected to this rebreathing valve was 6-foot hose attached to a 60-L Douglas bag filled with a hypercapnic gas mixture (5% CO_2_, 21% O_2_, balanced N2) (Liu et al., 2021; Liu et al., 2019). A nose clip was fitted to each participant to completely restrict nasal breathing, and they were instructed to breath normally through their mouth for the entirety of the scan. A lab member positioned next to the participant during the scan manually switched the 2-way valve between room air to the Douglas bag air mixture. Participants breathed normal room air for four 50s blocks interleaved with hypercapnic breathing for three 50s blocks. Tidal CO_2_ concentrations were assessed via a sampling line connected to the mouthpiece apparatus, which was fed out to an adjacent room containing a Philips NM3 Capnograph (Philips Respironics, Murrysville, PA, United States). Raw CO_2_ time series was sampled at rate of 100 Hz.

### 2.3 MRICloud pipeline

#### 2.3.1 Segmentation

To accurately quantify CVR at varying degrees of spatial granularity, multi-atlas segmentation of our anatomical images (T1w) was submitted to the fully automated MRICloud pipeline (www.mricloud.org) (Mori et al., 2016). Prior to this submission, our raw dicom files were converted to 4dAnalyze format using custom software provided by MRICloud. These converted files were then submitted to the pipeline for orientation and homogeneity correction, along with skull stripping procedures. Multiple atlases are then registered to this image using Large Deformation Diffeomorphic Metric Mapping (LDDMM) (Ceritoglu et al., 2010), followed by Diffeomorphic Multi-Atlas Likelihood Fusion (DMALF) (Tang et al., 2013). Based on our population of interest, we selected the “Adult_50_90yrs_289Labels_19atlases_M2_252_V7A″ atlas.

#### 2.3.2 End-tidal CO_2_ (etCO_2_) extraction

Both TDS and fMRI BOLD based CVR studies have established the benefits of characterizing CVR relative to change in, etCO_2_ (Hou et al., 2020). To derive, etCO_2_, the raw CO_2_ time series required export to. txt format using Philips custom software (Waveform Viewer). This raw data was then truncated (removing any data points not involving participant respiration before/after the scan) and linearly scaled and shifted via a Matlab-based code provided on MRI-cloud CVR v.5. This was done to ensure that only raw CO_2_ data recording participant breathing was used for subsequent, etCO_2_ extraction.

After these preprocessing steps, our truncated, scaled/shifted CO_2_ data was submitted to the MRICloud CVR. v5 processing pipeline. A comprehensive outline of the, etCO_2_ extraction steps from this pipeline is provided by Liu et al., 2022 (Liu et al., 2022). Briefly, etCO_2_ (the upper wave envelope of the raw CO_2_ time series) was extracted after data smoothing (100 m moving window), downsampling (10 Hz), peak identification, partial breath peak removal, and interpolation of peak-to-peak CO_2_ time course were performed. This, etCO_2_ time course is then temporally aligned with the BOLD-CVR time course through an iterated regression process. The time-shifted regression iteration with the lowest residual error was then used to optimize, etCO_2_ BOLD-CVR alignment.

The CVR. v5 pipeline also performs fMRI preprocessing including time-shift correction, motion correction and spatial smoothing (8 mm FWHM Gaussian Kernel) of the raw BOLD data using SPM12 (Friston et al., 2003). After completing BOLD preprocessing and temporal alignment procedures, CVR is computed using a generalized linear model (GLM) between the BOLD signals and time-shifted etCO_2_:

$$CVR=\frac{\beta_{1}}{\beta_{0}-\beta_{1}\cdot \left[mean\left(etCO_{2}\right)-baseline\left(etCO_{2}\right)\right]}\cdot 100$$ (1)

Where β1 and β_0_ are coefficient estimates to calculate CVR (%/mmHg), mean (etCO_2_) is the mean of the entire, etCO_2_ signal, and baseline (etCO_2_) is the mean of the lowest 25% of the, etCO_2_ signal. Note that this equation is done both globally (on the average whole-brain BOLD-CVR signal), and regionally according to our brain segmentation mapping steps. Regional CVR is calculated with the same Equation 1, however, additional, etCO_2_ alignment procedures are performed for each region prior to this calculation.

The MRICloud pipeline produces text-formatted output file from the above segmentation and CVR calculations. Based on the atlas used for segmentation, this output included CVR outcomes for the whole brain (grey matter) and three scales of ROI-specific CVR (19, 54, 289 ROIs).

In addition to the quality control processes embedded in the MRICloud pipelines, the following in-house steps were taken before and after submission to cortical segmentation and CVR pipelines. 1) Prior to submitting T1-MPRAGE data for segmentation, the raw dicoms were converted to NIfTI format and visually inspected using AFNI software (Cox, 1996). These data were only submitted to segmentation after they were inspected for signal homogeneity, motion artifacts, and head coverage. 2) Cortical segmentation data outputs were visually inspected using a graphical user interface embedded in the MRICloud website. Here, the T1 images are presented with cortical masks reflecting segmentation of 19, 54, and 289 regions of interest. All images were visually assessed for segmentation accuracy prior to submitting these data to the CVR pipeline. 3) Prior to submitting our raw BOLD-CVR data to the CVR pipeline, we converted our raw BOLD data to NIfTI format and visually inspected these images/time-series using AFNI. We examined signal quality (dropout, excessive equipment noise, signal drift), and checked for visual confirmation of three consecutive “peaks” in the BOLD time-series consistent with our hypercapnic challenge paradigm. Confirmation of CVR response was examined across voxels in the axial plane of the parietal lobe for all participants. 4) Visual inspection of, *etCO*_*2*_ data quality (signal dropout, drift) was also performed, along with confirmation of three “peaks” in the time-series before submission. 5) Included in the CVR output text files are whole-brain and regional cross-correlation coefficients values between BOLD and, *etCO*_*2*_. Participants included in our analysis exhibited a mean cross-correlation coefficient of 0.81 (±0.07).

### 2.4 Exercise testing

Cardiovascular fitness was assessed using the modified Balke submaximal graded exercise test (Balke and Ware, 1959; Eike et al., 2021) on a Woodway treadmill (Woodway, Waukesha, WI). Before testing, participants were fitted with a facemask that allowed them to breathe normally through their nose and mouth. A mass flow sensor was connected to this mask and fed to a Cosmed metabolic cart (Cosmed, Italy) to acquire breath-by-breath measures of minute ventilation (VE), oxygen uptake (VO_2_) and carbon dioxide production (VCO_2_). Heart rate was also continuously acquired using a chest strap monitor. Specifically, a Garmin HRM-dual heart rate monitor (Garmin Ltd., Olathe, Kansas) or smartLAB hrm monitor were used interchangeably (HMM Diagnostics GmbH, Heddesheim, Germany), as both units were provided by Cosmed and demonstrated between unit-reliability.

Testing began with an initial 2-min walk at 3.0 miles per hour (mph) and 0% treadmill grade. Treadmill grade was then incrementally increased by 2%–3% every 2 minutes until either a) participants discontinued the test due to exhaustion, or b) reached 90 of their estimated heart rate maximum (220-age). As soon as either condition a or b was met, treadmill speed was reduced to 2mph and grade to 0% for a 2-min cooldown. Raw breath-by-breath and heart-rate time series from GXT were then exported for subsequent processing and analysis using custom Matlab scripts written in-house (Mathworks Inc.). Interestingly, our analysis of heart rate data found that participants elected to discontinue GXT testing at an average of 89% of their estimated heart rate maximum.

Preliminary processing was performed on all breath-by-breath measures including absolute VO_2_ (mL/min), VO_2_ normalized to participant weight (mL/kg/min), VCO_2_ (mL/min), and VE (mL/min). These data were filtered using the Savitsky-Golay technique (Savitzky and Golay, 1964), where a moving-window least-squares regressions is performed. A second-order polynomial model was selected for data fitting, and we specified a window length of 29 data points to be used for each iterated regression. Breath-by-breath data from the cooldown phase of exercise testing was not included for processing or any subsequent calculations/analyses.

**FIGURE 1:**
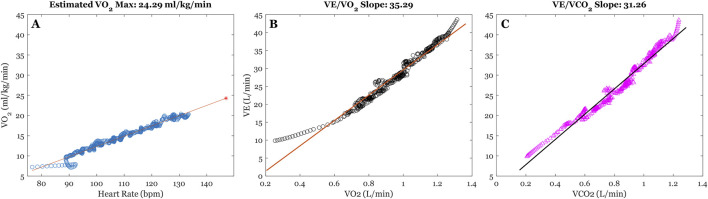
Illustration of time-series from graded exercise testing (GXT) for a sample participant. **(A)** Illustrates the least-squares regression between filtered Heart Rate (bpm) and VO_2_ (mL/kg/min) time-series to extrapolate VO_2_ max estimates. The blue dots reflect filtered time-series, the orange line reflects the least-squares fitting, and the red star represents estimated VO_2_ max at individuals HR max (220-age). **(B,C)** illustrates the least-squares regressions between minute ventilation (VE (L/min)), absolute VO_2_ (L/min), and absolute VCO_2_ (L/min), respectively. The estimated slope parameters from model fitting us used as our indices of ventilatory efficiency.

To index cardiovascular fitness, we calculated participants estimated VO_2_ max (mL/kg/min) based on values attained during GXT (Wasserman, 1984). Specifically, a least-squares linear regression was calculated between Savitsky-Golay filtered Heart Rate and VO_2_(mL/kg/min) time-series during the exercise phase of GXT. Participants estimated VO_2_ max was extrapolated as the predicted VO_2_ value that intersects with their estimated heart-rate maximum (220-age) (Figure 1A) (Lee et al., 2011).

We also examined ventilatory efficiency during graded exercise, which encompasses the relation between change in minute ventilation (VE) and changes in oxygen uptake (VO_2_ (L/min) and carbon dioxide elimination (VCO_2_ (L/min)) (Phillips et al., 2020) (see Phillips et al., 2020 for a comprehensive review on measurement and interpretation). Specifically, we performed separate least-squares linear regression between VE/VO_2_ and VE/VCO_2_ time-series, and the estimated slope parameters from these regressions were used to index ventilatory efficiency.

### 2.5 Statistical analyses

Matlab version 2024a (the Mathworks Inc., Natick, Massachusetts) was used to conduct data processing, visually inspect MRICloud CVR output data and GXT data, and calculate CVR and GXT outcomes. From our preprocessing, three subject data points were removed from any statistical analyses. One participant was removed due to signal dropout during GXT, and two were removed due to dropout of, etCO_2_ signal during the CVR scan. This was done to mitigate the potential for inaccurate CVR calculation, and thus data from 20 participants (14 female) was used for our final analyses.

Multiple linear regression (MLR) was performed to examine the degree of variance in whole-brain CVR that is accounted for by the fitness variables VO_2_ max (mL/kg/min), VE/VCO_2_ slope, and VE/VO_2_ slope. The fitted model is shown in Equation 2:

$$CVR=A+B\cdot {VO}_{2}\max+C\cdot {\frac{VE}{VCO}}_{2}slope+D\cdot {\frac{VE}{VO}}_{2}slope+e$$ (2)

Where *A, B, C, D* represent fitted regression parameters, and *e* is the error term. This analysis was performed using JMP Pro statistical software (JMP Pro, version 16, SAS Institute Inc., Cary, NC, 1989–2024) using a standard least-squares fitting. Results of the whole model fit (*F* and associated *p-value*) are reported in the results section, and results of fit for each parameter (*t* and associated *p-value*, confidence intervals, and collinearity diagnostics (Variance Inflation Factor (VIF)) are provided in Table 2.

As an exploratory component of this study, we used custom Matlab scripts to run separate Pearson’s correlations between fitness outcomes and CVR based on 64 ROI’s (32 per hemisphere) derived from the 289 ROI cortical segmentation atlas. To attain CVR outcomes for our regions of interest, we averaged CVR across any gyri that collectively corresponded with a whole region of interest (i.e., Inferior Frontal Gyrus (IFG) CVR = average of IFG Opercularis CVR, IFG orbitalis CVR, and IFG triangularis CVR). Due to the descriptive nature of this ROI-specific analysis, no corrections for multiple comparisons were applied. Plotting of fitness and ROI-specific CVR correlations was done using custom Matlab scripts.

## 3 Results

Participant demographics from our population sample are shown in Table 1.

**TABLE 1:** Participant demographics.

Demographic	Mean (range)
Sex (M/F)	7/16
Age (years)	70.91 (65–79)
Education (years)	17.15 (13–22)
Estimated VO_2_ max (mL/kg/min)	21.84 (12.90–31.91)
CVR: ΔBOLD (%)/ΔetCO_2_ (%)	12.51 (5.68–27.98)
VE/VCO_2_ slope	31.68 (25.00–38.37)
VE/VO_2_ slope	38.28 (26.95–54.66)

**FIGURE 2:**
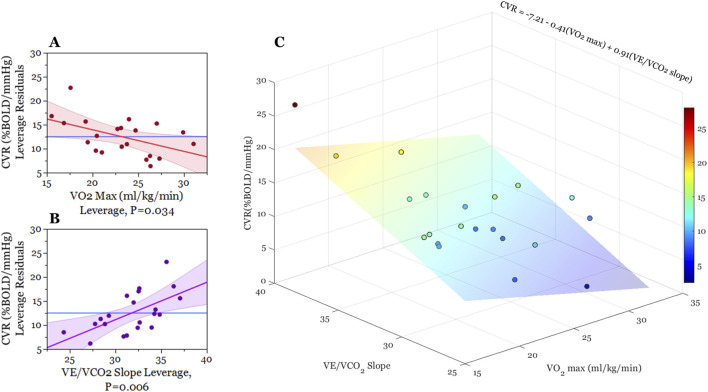
Graphical illustration of multiple regression analysis results. **(A,B)** are partial regression leverage plots illustrating the effects of each term in the model (2a: VO_2_ max, 2b: VE/VCO_2_) while controlling for the effects of all other predictor variables (VO_2_ max, VE/VO_2_, VE/VCO_2_). On the y-axis, whole-brain CVR values are regressed on all predictor variables except for the predictor term in question. The mean of whole-brain CVR is then added to these residuals. For 2a and 2b x-axes, respectively, VO_2_ max and VE/VCO_2_ slope are regressed on all the other predictor variables and then mean VO_2_ max and mean VE/VCO_2_ slope are added to these residuals. The dots on each plot reflect these x- and y-leverage pairs. The blue line (mean BOLD-CVR) represents the null hypothesis that CVR is independent of VO_2_ and VE/VCO_2_ slope, while the red and purple lines represent least-squares fitted line between the specified x-y leverage pairs (along with confidence bands of the slope estimate). The non-zero slope of the red and purple lines demonstrate that adding VO_2_ max and VE/VCO_2_ slope estimates to the model (Table 2) account for significant variance in BOLD-CVR outcomes. **(C)** is a three-dimensional scatter plot illustrating the relationship between VO_2_ max, VE/VCO_2_ slope, and BOLD cerebrovascular reactivity. Each point represents an individual participant, and the color of these points correspond with whole-brain CVR. For the sake of illustration, we created a least-squares regression plane indicating the best-fit model predicting cerebrovascular reactivity from VO_2_ max and VE/VCO_2_ slope outcomes. This regression plane did not include a term for VE/VO_2_).

Our multiple regression model demonstrated statistical significance (F (3,17) = 7.33, *p =* 0.002), accounting for 56% of the variance in whole-brain CVR. When controlling for the effects of all fitness variables, we found that estimated VO_2_ max (mL/kg/min) (*t =* −2.30*, p =* 0.03) and VE/VCO_2_ slope (*t =* 3.12*, p =* 0.00) outcomes were significant in describing CVR across the cortex (as seen in Figure 2; Table 2). Individuals with a higher VO_2_ max estimate and lower VE/VCO_2_ slope (indicating greater ventilatory efficiency) showed a lower CVR response to hypercapnia relative to individuals with lower fitness. Moreover, the VE/VO_2_ slope term did not have a significant effect on CVR within our model (*t =* −0.97*, p =* 0.35).

**TABLE 2:** Multiple regression model relating cardiovascular fitness measures to whole-brain CVR.

Parameter	β	Std. Err	t	*p*	95% CI	VIF
Intercept	3.07	10.84	0.28	0.78	−19.81/25.94	
VO2 Max	−0.45	0.19	−2.30	0.034*	−0.86/-0.-38	1.118
VE/VO2 Slope	−0.13	0.13	−0.97	0.35	−0.40/0.15	1.0333
VE/VCO2 Slope	0.78	0.25	3.11	0.006**	0.25/1.31	1.1531

β denotes the model coefficient, Std. err denotes the standard error of model coefficients, t denotes the t-statistic, p denotes the alpha threshold, 95% CI, denotes a two-sided confidence interval of model coefficients, VIF, denotes the variance inflation factor testing for collinearity (1.0 = zero collinearity, >5 significant collinearity). *and ** indicate significance at p ≤ .05. Text in bold indicates statistical significance.

**FIGURE 3:**
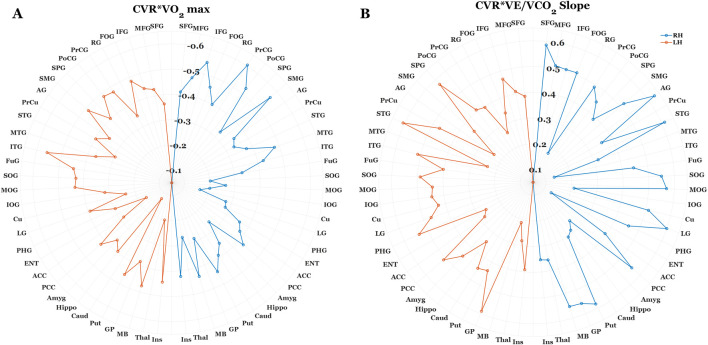
Polar plots illustrating the correlation strength between regional BOLD-CVR, Estimated VO_2_ max **(A)**, and VE/VCO_2_ slope **(B)**. Each data point corresponds with the correlation coefficient (R) between fitness outcomes and CVR at a specific region of interest (ROI). The orange and blue dotted lines correspond with R values for the left and right brain hemispheres, respectively. For the sake of clarity, 63 of the 289 ROI’s were included in this analysis. In cases where cortical segmentation produced more than one CVR value within-gyri (I.e., Inferior frontal gyrus opercularis/orbitalis/triangularis), these values were averaged prior to correlation analysis. For both fitness outcomes, there was a consistent relation between lower CVR and higher fitness levels. However, correlation strength was notably higher between BOLD-CVR and Estimated VO_2_ max at regions within the frontal, temporal, and parietal cortices. Conversely, the correlation strength between BOLD-CVR and VE/VCO_2_ slope were homogenous across cortical regions.

In accordance with our multiple regression results, we used correlation analysis to explore whether VO_2_ max estimates and VE/VCO_2_ slope demonstrate regional differences on their relation to CVR. Figure 3. Illustrates this exploratory analysis using polar plots (3a. CVR x VO_2_ max; 3b CVR x VE/VCO_2_). This analysis revealed a consistent trend between lower CVR and higher fitness across the entire cortex, meaning we observed no instances where higher regional CVR related to better fitness. Moreover, this inverse correlation strength with VO_2_ max was consistently higher in sub-regions of the frontal, temporal, and parietal lobes relative to all other regions of interest. Conversely, we observed a pattern of homogeneity in correlation strength with VE/VCO_2_ slope and CVR across all cortical ROI’s.

## 4 Discussion

Low cardiovascular fitness levels in older adults is a well-recognized risk factor for numerous adverse health outcomes, including impaired cardiovascular function and diminished cerebral blood flow regulation (Falck et al., 2017; de Rezende et al., 2014). While this general association is well established, the specific mechanisms through which sedentary behavior influences aging trajectories remain poorly understood. This gap in understanding is partly due to the highly individualized nature of functional decline among older adults. Variability in this decline may stem from a complex interplay of factors, such as genetic predisposition or prior physical activity history, each of which influences cardiorespiratory fitness across the lifespan (Bye et al., 2020; Carrick-Ranson et al., 1985; Lavie et al., 2019). Given this complexity, the current study aimed to investigate whether fitness levels, particularly markers related to ventilatory and perfusion-based regulation of oxygen uptake and carbon dioxide elimination, significantly modulate CVR in older adults.

Our findings demonstrated that higher cardiovascular fitness (higher estimated VO_2_ max, and lower VE/VCO_2_ slope) during graded exercise was associated with lower whole-brain BOLD-CVR responses (grey matter) to hypercapnic challenge. Of note, the strength of the relationship between VO_2_ and BOLD-CVR was increased in the frontal, temporal, and parietal lobes relative to all other regions of interest. Interestingly, the correlation strength of BOLD-CVR with VE/VCO_2_ slope was consistent across all ROI’s. Also of note, the mean and standard deviation of CVR values observed in our cohort are consistent with values observed in previous fMRI-based aging and CVR studies (12.51 ± 5.00) (Lu et al., 2022). Additionally, these values are comparatively lower than those observed in young healthy individuals (Lu et al., 2022; Leoni et al., 2017).

### 4.1 CVR fitness relations across imaging modalities

Our results challenge the overarching perception that higher CVR is a robust index of a healthier brain. This is primarily supported by transcranial Doppler (TDS) studies showing that higher CVR is associated with better fitness (Barnes et al., 2013), and TDS and fMRI studies showing a consistent association between lower CVR and normal aging (Kastrup et al., 1998). Despite their complementary findings, and the significance of hemodynamic response to hypercapnia in both TDS and fMRI signals, our findings highlight the need for caution when comparing CVR across these imaging modalities. This is supported by recent work showing no clear relationship between BOLD- and TDS-derived CVR within healthy younger and older adults (Burley et al., 2021). This discrepancy may stem from each method capturing different vascular components: TDS measures blood flow velocity in large arteries (e.g., MCA), whereas BOLD reflects changes in deoxyhemoglobin in venous vessels, influenced by blood volume, arteriolar flow, and oxygen metabolism (Buxton et al., 1998).

Increased TDS-CVR is often interpreted as beneficial, as faster flow in major arteries supports downstream neurovascular coupling. BOLD-CVR, however, may also encompass fitness-related shifts in cerebral metabolism (cerebral metabolic rate of oxygen: CMRO_2_). While hypercapnic paradigms assume stable cerebral metabolism, studies report mixed CMRO_2_ responses—some unchanged (Chen and Pike, 2010), some reduced (Peng et al., 2017; Xu et al., 2011), and others elevated (Jones et al., 2005). More recently, dynamic systems modeling using fNIRS and TDS data suggests hypercapnia increases blood flow while suppressing cortical oxidative metabolism, likely via mitochondrial signaling pathways (Highton et al., 2024). The effects of cerebral metabolism may a) account for the disparate findings from TDS/fMRI imaging modalities, and b) further explain variance in BOLD-CVR response to hypercapnia with aging and fitness, however, confirmation of these effects will require multi-modal imaging in future work.

### 4.2 VO_2_ and VE/VCO_2_ in relation to CVR

To our knowledge, this is the first study to demonstrate a significant association between greater ventilatory efficiency (VE/VCO_2_) and lower BOLD-assessed CVR in older adults. Our results also provide empirical support for an inverse relation between VO_2_ outcomes and CVR observed in previous imaging studies (Intzandt et al., 2019; Thomas et al., 2013a). Thomas et al. (2013), for example, observed lower hypercapnic CVR in master’s athletes relative to age-matched sedentary, and young healthy adults. They also found a significant inverse relation between peak VO_2_ and CVR outcomes within their sample of master’s athletes, thus indicating a potential dose-response relationship between fitness and cerebral reactivity. Our findings extend this possibility to broader aging populations, although examining factors such as lifelong physical activity and genetics in mediating individual cardiorespiratory and cerebrovascular health across a range of aging subpopulations is warranted.

Nevertheless, the physiological rationale behind higher fitness and lower BOLD-CVR has been primarily attributed to reduced sensitivity to systemic CO_2_ levels with greater physical activity. The basis of this assumption stems from previous work showing that submaximal aerobic exercise may induce elevated carbon dioxide levels, chronically, in the cerebrospinal fluid (Intzandt et al., 2019; Thomas et al., 2013b). Numerous physiological mechanisms, potentially working in concert, may help to explain our findings and the underlying relation between physical fitness and cerebrovascular health, which are discussed below.

#### 4.2.1 Improved cardiorespiratory efficiency and reduced hypercapnic response

Higher VO_2_ is a hallmark of aerobic capacity, driven by increased oxygen delivery and utilization, enhanced capillary and mitochondrial density, and oxidative enzyme activity (Booth et al., 2015). These adaptations may reduce the need for vasodilatory responses to CO_2_ during hypercapnia (Thomas et al., 2013a).

#### 4.2.2 VE/VCO_2_ Slope and respiratory stability

A lower VE/VCO_2_ slope reflects efficient CO_2_ ventilation, improved pulmonary exchange, and buffering capacity (Chua et al., 1997). It also may indicate reduced chemosensitivity to CO_2_, leading to lower CVR (Ogoh and Ainslie, 1985), and more stable CO_2_ levels, reducing cerebrovascular fluctuations (Fisher et al., 1985).

#### 4.2.3 Integrated cardiovascular-cerebrovascular mechanisms

Higher VO_2_ max and lower VE/VCO_2_ slope are indicators of integrated cardiovascular and cerebrovascular adaptations. Improved cardiovascular fitness contributes to enhanced cardiac output, reduced arterial stiffness, and better endothelial function, all of which benefit cerebral perfusion and reduce the need for reactive increases in blood flow (Bailey et al., 2013). Additionally, these fitness markers reflect systemic improvements in oxygen and CO_2_ transport and utilization, leading to a more stable cerebrovascular environment.

Overall, the combination of high VO_2_ max and low VE/VCO_2_ slope reflects a comprehensive set of adaptations that may enhance oxygen transport, CO_2_ clearance, and blood flow regulation, which may result in lower whole-brain CVR. This integration suggests that fitter individuals may maintain cerebrovascular health and function through multiple interrelated mechanisms that, while compensatory, potentially further decrease BOLD-CVR outcomes in parallel with aging-related changes in cerebrovascular health. To reiterate, direct evaluation of these mechanistic underpinnings will require multi-modal imaging to further inform the dynamic effects of aging and fitness on cerebrovascular health.

### 4.3 Regional CVR related differently with VO_2_ and VE/VCO_2_ outcomes

Despite better fitness showing a consistent trend with lower CVR across the entire cortex, there were distinct patterns of correlation strength between regional reactivity and estimated VO_2_ max *versus* VE/VCO_2_ outcomes. Specifically, CVR in gyri within the frontal, temporal, and parietal lobes demonstrated a stronger correlation strength to VO_2_ outcomes, whereas the correlation strength with VE/VCO_2_ was both strong and consistent across all cortical regions of interest (see Figures 3A,B). Collectively, this highlights that these distinct cardiorespiratory adaptations may exhibit distinct contributions to local *versus* global cerebrovascular health with aging. As discussed in the previous section, VO_2_ primarily underscores fitness adaptations in the context of efficient delivery/utilization of oxygen (Snell and Mitchell, 1984), which is a critical factor for maintaining metabolic needs to facilitate effective neurotransmission (Salzman et al., 2022). That VO_2_ adaptations have a stronger contribution to the regions that are a) particularly vulnerable to aging effects, and b) have significant capillary density and are more metabolically demanding due to their significance to cognitive and sensorimotor functions (Cabeza and Dennis, 2012; Harrison et al., 2002; Jack et al., 1998; Oedekoven et al., 2013), it suggests greater systemic metabolic efficiency may help to account for a less pronounced hypercapnic cerebrovascular response in these areas. Interestingly, this may complement previous work showing that lower VO_2_ outcomes relate to patterns of BOLD hyperactivation in the frontal cortices during cognitive tasks in older adults (McGregor et al., 2013; Nocera et al., 2017). In contrast to VO_2_, greater ventilatory efficiency (lower VE/VCO_2_ slope) and its association with lower whole brain CVR may reflect adaptations in CO_2_ regulation regardless of differing vascular and metabolic demands across the cortex. The functional significance of both VO_2_ and VE/VCO_2_ outcomes suggests that multimodal assessment of cardiorespiratory fitness can also improve our mechanistic understanding of cerebrovascular health with aging and exercise.

### 4.4 Limitations and future directions

A primary limitation to the present study is our limited sample size. While this constrains on our capacity to draw conclusive inferences about the aging population, our VO_2_*BOLD-CVR relations are consistent with previous fMRI studies, and thus these findings may help inform future work aiming to elucidate the mechanistic underpinnings, that can explain CVR dynamics, specific to various vascular bedding, as a function of aging and exercise.

A second limitation on the interpretability of this study, and a general limitation within the CVR literature, is the variability in vasoactive stimulus to induce vascular reactivity. At present, the primary approaches in both TDS and fMRI studies are to induce hypercapnic conditions through breath holding (Urback et al., 2017), hyperventilation (Boulet et al., 2016), and breathing fixed amounts of CO_2_ (Liu et al., 2019). Of these techniques, fixed CO_2_ inhalation is the most common approach due to its low equipment cost and ease of application. Specific to CO_2_ inhalation, it is argued that we’re indirectly measuring PaCO_2_ based on estimates of, etCO_2_, however, this relation is not necessarily linear, particularly in populations outside of the young healthy population (Fierstra et al., 2013b). As such, there is recent work suggesting that the use of computerized gas mixtures to maximize, etCO_2_ response at the individual level, thus providing an accurate representation of the boundary conditions of one’s CVR capacity.

Our exploratory analysis requires a larger sample size to run specific statical analyses that can help predict the functional significance of cardiovascular fitness on ROI-specific CVR outcomes (I.e., draw inferences on how this relation may help to explain potential behavioral changes to cognition and sensorimotor function). The goal was to better describe the patterned regional differences in CVR/fitness relations. In doing so, we emphasize the significance of better understanding both between- and within-subject differences in cerebral vascular functions. That is, regression analyses showed that spatial homogeneity in CVR existed with respect to between-subject CVR differences (the trend of better fitness corresponding with lower CVR was found across all ROI’s). That said, the fact this relation to estimated VO_2_ max outcomes was clearly stronger in the cortices susceptible to aging-related declines points the potential richness of information from within-subject fluctuations of CVR. Nevertheless, translation of these findings requires a larger sample size and subsequent statistical corrections (I.e., adjustment for multiple comparisons).

While our results outline the need to better derive physiological adaptations that a) contribute to variability in BOLD-CVR signal, and b) help to explain the relation between higher fitness and lower CVR in our older adults, it is important to note that multi-modal imaging is necessary to directly estimate the potential mechanistic underpinnings outlined in discussion sections 4.1–4.3. As such, future research including BOLD-CVR with TDS to examine dynamics between arterial and venous CVR, Magnetic Resonance Spectroscopy (MRS) and fNIRS to examine changes in central and peripheral oxidative capacity (Marusic et al., 2019; Ryan et al., 2013), calibrated BOLD and/or TRUST MRI (T2-relaxation-under-spin-tagging) to quantify changes in cerebral metabolic rate (Chen et al., 2022; Lu et al., 2012), are just a few methods that can be used in conjunction with BOLD-CVR and O_2_/CO_2_ kinetics during exercise to further inform results from the current study. Lastly, there is evidence that hypercapnic and hypoxic gas mixtures may individually alter CMRO_2_, however, a hypercapnic-hypoxic gas challenge may stabilize cortical metabolism and elicit an even greater CBF response compared to hypercapnia alone (Ravi et al., 2016). Thus, examining this approach, particularly in combination with multi-modal imaging, may be warranted.

Additional limitations of our study can be seen in utilizing a submaximal GXT along with our HR max calculation. Our protocol required we discontinue the V02 assessment once the participants reached 90% HR max. Using 220-age to estimate HRmax likely underestimated HRmax and may have resulted in the test being discontinued before participants reached their “true” 90% HR max. However, given the nature of the sedentary cohort, most discontinued the exercise prior to achieving our calculated HR max and thus we feel this had limited impact our on findings. While more accurate equations exists, it remains the case that whatever equation is used, there is variation of max HR with age calculations, particularly in aging.

From this work, we propose that an important future research direction is examining the role of individual fitness, specifically in terms of ventilatory efficiency, as it relates to cerebrovascular health in various clinical populations. Accurate calculation of ventilatory efficiency does not require near maximal exercise, and thus this is a much more viable paradigm for populations that cannot meet near maximal exercise requirements necessary to attain reliable VO_2_ estimates. Moreover, VE/VCO_2_ has shown tremendous prognostic potential in predicting functional and mortality outcomes in populations such as chronic heart failure (Poggio et al., 2010), and COPD (Gonzalez-Garcia et al., 2021).

## Data Availability

The raw data supporting the conclusions of this article will be made available by the authors, without undue reservation.
